# Supplementary material for: The interactome of intact mitochondria by cross-linking mass spectrometry provides evidence for coexisting respiratory supercomplexes
Source: Mol Cell Proteomics. 2017 Dec 8;17(2):216–32. doi: 10.1074/mcp.RA117.000470 (PMC5795388; doi:10.1074/mcp.RA117.000470)
Supplement: Supplemental Data [file supp_RA117.000470_133922_0_supp_23978_fzffwf.zip › spectra_annotation/mito_DR_spectra_annotation/411-1-14-1-9-1.pdf]

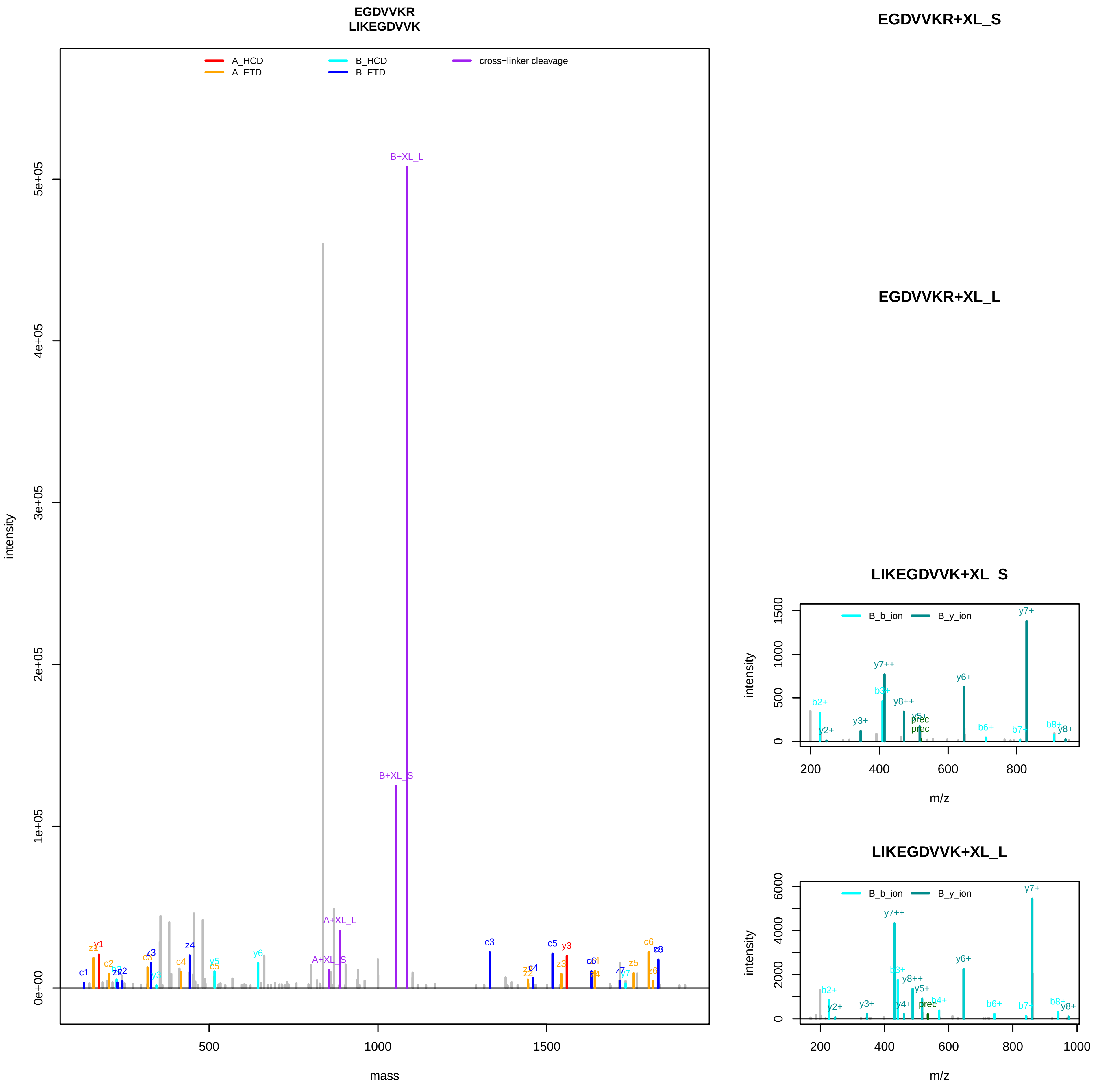

EGDVVKR
LIKEGDVVK
A_HCD
A_ETD
B_HCD
B_ETD
cross−linker cleavage
B+XL_L
B+XL_S
A+XL_L
A+XL_S
intensity
mass
0e+00
1e+05
2e+05
3e+05
4e+05
5e+05
500
1000
1500
EGDVVKR+XL_S
EGDVVKR+XL_L
LIKEGDVVK+XL_S
LIKEGDVVK+XL_L
B_b_ion
B_y_ion
m/z
prec
